# The impact of CYP2D6*41 on *CYP2D6* enzyme activity using phenotyping methods in urine, plasma, and saliva

**DOI:** 10.3389/fphar.2022.940510

**Published:** 2022-08-30

**Authors:** Ye Jin, Shuquan Zhang, Pei Hu, Xin Zheng, Xiaoduo Guan, Rui Chen, Shuyang Zhang

**Affiliations:** ^1^ State Key Laboratory of Complex Severe and Rare Diseases, Peking Union Medical College Hospital, Chinese Academy of Medical Science and Peking Union Medical College, Beijing, China; ^2^ Chinese Academy of Medical Science and Peking Union Medical College, Beijing, China; ^3^ Clinical Pharmacology Research Center, Peking Union Medical College Hospital, Chinese Academy of Medical Sciences and Peking Union Medical College, Beijing, China; ^4^ Department of Cardiology, Peking Union Medical College Hospital, Chinese Academy of Medical Sciences and Peking Union Medical College, Beijing, China

**Keywords:** CYP2D6*41, phenotyping method, metabolic ratio, polymorphism, genotype

## Abstract

**Aims:** The CYP2D6*41 variant is the second or third frequent reduced function allele in Chinese with a frequency of around 3–4%, while it is the major reduced function allele in Indians, Saudi Arabians and Caucasians with frequencies of around 10–20%. The present study was designed to explore the impact of CYP2D6*41 on the metabolic activity of *CYP2D6* using phenotyping methods in urine, plasma, and saliva.

**Methods:** We used dextromethorphan as the probe drug to analyze the phenotypes of 87 subjects with CYP2D6*1/*1 (*n* = 22), CYP2D6*1/*2 (*n* = 33), CYP2D6*2/*2 (*n* = 4), CYP2D6*1/*41 (*n* = 5), CYP2D6*2/*41 (*n* = 3), CYP2D6*10/*41 (*n* = 16), and CYP2D6*5/*41 (*n* = 4) for *CYP2D6*. The ratio of parent drug to metabolite in 3 h saliva, 3 h plasma, and in 0–3 h urine was considered the metabolic ratio (MR).

**Results:** The CYP2D6*41 allele had substantial impact on the metabolic activity of *CYP2D6* regardless of the urinary, plasma, or salivary phenotyping method used. In subjects with CYP2D6*1(or *2)/*1(or *2), *1 (or *2)/*41, *10/*41 and *5/*41 (all *p* < 0.001), the salivary, plasma, or urinary MR value increased. The MRs in saliva, plasma, and urine displayed high correlations.

**Conclusion:** The activity score system or the consensus activity score system, instead of the traditional phenotype classification, could predict the *CYP2D6* enzyme activity more accurately. CYP2D6*41 had similar or more impact on the *CYP2D6* enzyme activity as compared with CYP2D6*10. Assigning *41 a score of 0.5 and assigning *10 a score of 0.25 according to the consensus AS system should be reconsidered.

## Introduction

Cytochrome P450 2D6 (*CYP2D6*) is directly involved in the metabolism of approximately 25% of currently approved medications, including antidepressants, antipsychotics, analgesics and antitussives, beta adrenergic blocking agents, antiarrhythmic, antiemetics, etc. (Zhou, 2009a; Zhou, 2009b; Murphy and McMahon, 2013; Zanger and Schwab, 2013). *CYP2D6* polymorphisms have been extensively investigated in terms of their effects on enzymatic activity. Differences in *CYP2D6* activity are derived from over 100 variants (The Pharmacogene Variation Consortium; https://www.pharmvar.org/gene/CYP2D6) (Zanger et al., 2004; Teh and Bertilsson, 2012). Among these are fully functional alleles, reduced function alleles, nonfunctional alleles and gene copy duplicates, that range in activity from ultra-rapid metabolism to no metabolism (Gaedigk, 2013; Kevin Hicks et al., 2016; Gaedigk et al., 2017).

Previous studies have revealed significant ethnic and geographic differences in the frequencies of *CYP2D6* alleles (Bradford, 2002). CYP2D6*10 is predominant in Asian populations, with the frequency ranging from 30 to 50% (Qian et al., 2013). CYP2D6*4 distinguishes Caucasians from other populations with a frequency of 12–21% (Qian et al., 2013). The frequency of CYP2D6*41 was reported to be 3.05% in Chinese (Qian et al., 2013), while it was identified as the major reduced function allele in Indian population with a frequency of 12.3% (Manoharan et al., 2019). High prevalence of CYP2D6*41 was also reported in Saudi Arabians and Caucasians with frequencies of 18.4% (Al-Dosari et al., 2013) and 11.1% (Dalton et al., 2020), respectively.

In clinical settings, *CYP2D6* phenotypes are usually classified into metabolizer groups, like the following: ultra-rapid (UM), normal (NM), intermediate (IM), and poor (PM) (Zanger et al., 2004). Probe drugs are used to analyze the metabolic activity of *CYP2D6*, with the most-used substrate being dextromethorphan (DM) (Chen et al., 2017). The metabolic ratio (MR) of DM to its metabolite dextrorphan (DX) in 8-h urine after dosing is widely used to differentiate NMs from PMs (Chladek et al., 2000; O’mathúna et al., 2008; Lötsch et al., 2009; Ito et al., 2010). Collecting urine at 8 h intervals is not feasible in clinical settings. As such, alternative techniques are used for fast and efficient phenotyping. Saliva and plasma samples can also be used to analyze the MR value. Chladek et al. reported a close correlation between the MRs measured using urine (0–4 h post-dose) and plasma (3 h post-dose) [16]. Additionally, MR from single-point plasma collected 1–30 h post-dose displayed a positive correlation with MR from area under the curve (AUC) (Chen et al., 2016a; Chen et al., 2016b). In addition, the MR from 0–3 h urine, 3 h single-point plasma, and 3 h single-point saliva were reported to have good correlations and could all be used as alternative methods to determine the phenotype of *CYP2D6* (Chen et al., 2017).

Clinical Pharmacogenetics Implementation Consortium (CPIC) and Dutch Pharmacogenetics Working Group (DPWG) recently reported some discrepancies in their guidelines, primarily relevant to how certain *CYP2D6* genotypes were translated into phenotypes (Caudle et al., 2019). Gaedigk et al. introduced the activity score (AS) system for *CYP2D6* in 2008 (Gaedigk et al., 2008). Based on this system, a consensus definition method was also developed for more accurate “activity scores” (Caudle et al., 2019). In both systems, a value is assigned to each allele that designates its function. The total value of individual allele values is considered the final AS. It is well known that the CYP2D6*41 variant is a reduced function allele that decreases enzyme activity and thus increases the *CYP2D6* MR value. In the both systems, CYP2D6*41 variant is assigned a value of 0.5. Quantitative analysis on the how CYP2D6*41 affects different combinations of alleles has not been reported in relatively large populations. In the present study, CYP2D6*1 or *2 was defined as a standard full functional allele and CYP2D6*5 was defined as a nonfunctional allele. In addition, CYP2D6*10, with the very high frequency in Chinese, was defined as a reduced function allele. CYP2D6*1, *2, *5, and *10 were all used as references or control alleles to investigate how CYP2D6*41 affects different combinations of alleles. In the present study, salivary, plasma, and urinary phenotyping methods were used to analyze the activities of the *CYP2D6* enzyme in a healthy Chinese population. The results were then compared.

## Materials and methods

### Study subjects

The clinical study was carried out in accordance with the Guidelines for Good Clinical Practice and the Declaration of Helsinki. The clinical protocol was reviewed and approved by the Ethical Committee of Peking Union Medical College Hospital, Beijing, China. Each of the participants signed an informed consent form prior to enrollment. Four hundred and twenty-one subjects were enrolled in the study. After a detailed physical examination including routine urinalysis, hematology, biochemistry, and 12-lead electrocardiography, the subjects were declared healthy. Subjects were declared ineligible if they had the following: a history of hematologic, gastrointestinal, renal, or hepatic abnormalities, a human immunodeficiency virus, syphilis, hepatitis C or B, any chronic or acute disease, or were allergic to dextromethorphan. Consuming grapefruit juice, caffeinated beverages, or alcohol was not allowed in the 24 h before DM administration, nor until all samples were collected. Subjects were also instructed to refrain from ingesting herbal remedies or medication for a minimum of 1 week before the study, and to refrain from smoking for a minimum of 3 days before the study.

### *CYP2D6* genotyping

We analyzed the DNA sequence, by genotype, of each subject in the study for CYP2D6*1, *2, *3, *4, *6, *7, *10, *14, *18, *21, *28, *33, *34, *35, *36, *39, *41, *43, *49, *51, *52, *54, *60, *63, *65, *69, *71, and *75 using a previously reported method (Qian et al., 2013; Chen et al., 2017).

### *CYP2D6* phenotyping with DM

We used DX as the *CYP2D6*-specific metabolite and DM as the probe to analyze the phenotypes of 87 subjects with CYP2D6*1/*1 (*n* = 22), CYP2D6*1/*2 (*n* = 33), CYP2D6*2/*2 (*n* = 4), CYP2D6*1/*41 (*n* = 5), CYP2D6*2/*41 (*n* = 3), CYP2D6*10/*41 (*n* = 16), and CYP2D6*5/*41 (*n* = 4). We provided each subject with 15 mg of DM (Tylenol Cold Tablet containing DM, Johnson & Johnson Investment Ltd., Shanghai, China), along with 300 ml of water. Samples of saliva and venous blood were collected 3 h after the drug was administered, while samples of urine were collected at intervals from 0 to 3 h after the drug was administered. We analyzed DM concentrations and unconjugated DX in all of the samples using a validated and sensitive high-performance liquid chromatography tandem mass spectrometry (HPLC-MS/MS) assay, according to methods previously used [24, 25]. For DX and DM in the saliva, plasma, and urine samples, the lower limit of quantification was 0.05 ng/ml [15]. In order to measure the *CYP2D6* activity in the three types, a metabolic ratio of the concentration of DM over DX (MR_DM/DX_) was used.

### Statistical analysis

Data used were mean values ± SD. An analysis of variance (ANOVA) test was used to analyze the MR values between different groups of genotypes, based on the salivary, plasma, and urinary phenotyping methods. Alleles CYP2D6*2 and *1 were considered standard and fully functional, CYP2D6*5 was considered a nonfunctional allele, CYP2D6*10 was defined as a reduced function allele. All CYP2D6*1, *2, *5, and *10 were considered control alleles, allowing us to analyze the effects of the CYP2D6*41 allele on the metabolic activity of *CYP2D6*. In the original activity score system (Gaedigk et al., 2008), sore of 0 was assigned to CYP2D6*5; score of 0.5 was assigned to CYP2D6*10 and *41; score of one was assigned to CYP2D6*1 and *2. In the consensus activity score system (Caudle et al., 2019), sore of 0 was assigned to CYP2D6*5; score of 0.25 was assigned to CYP2D6*10; score of 0.5 was assigned to CYP2D6*41; score of one was assigned to CYP2D6*1 and *2. The score of a genotype was the sum of the values assigned to each allele in both of the systems. A *p* value less than 0.05 was considered to be statistically significant. The ANOVA was performed with SPSS (version 19.0, SPSS™).

## Results

### *CYP2D6* genotypes and demographic characteristics

The gene frequency of CYP2D6*41 was 3.56% among the 421 subjects. Twenty-two subjects had CYP2D6*1/*1, 33 subjects had CYP2D6*1/*2, 4 subjects had CYP2D6*2/*2, 5 subjects had CYP2D6*1/*41, 3 subjects had CYP2D6*2/*41, 16 subjects had CYP2D6*10/*41, and 4 subjects had CYP2D6*5/*41. The CYP2D6*41 allele was also observed in 2 gene duplications which were not reported in this study. We also tested additional *CYP2D6* alleles to assess the particulars of CYP2D6*1, *2, *5, *10 and *41.

Subjects with CYP2D6*1/*1, *1/*2, and *2/*2 were classified as the wild type. All 87 subjects’ ages, weights, body mass index (BMI) and genders can be found in Table 1.

**TABLE 1:** Demographic characteristics of the study population of healthy Chinese volunteers (mean ± SD).

Characteristic	All (n = 87)	Wild Type (CYP2D6*1/*1, *1/*2, and *2/*2) (as = 2, CAS = 2) (n = 59)	CYP2D6*1/*41 and *2/*41 (as = 1.5, CAS = 1.5) (n = 8)	CYP2D6*10/*41 (as = 1,CAS = 0.75) (n = 16)	CYP2D6*5/*41 (as = 0.5, CAS = 0.5) (n = 4)
Age (years)	30.4 ± 8.3	30.9 ± 8.2	26.6 ± 4.6	29.4 ± 8.2	34.0 ± 14.7
Weight (kg)	66.9 ± 8.5	67.3 ± 8.8	67.3 ± 6.1	66.3 ± 9.0	63.6 ± 6.9
BMI (kg/m^2^)	23.7 ± 2.4	24.0 ± 2.5	23.5 ± 2.4	22.8 ± 2.2	22.9 ± 1.6
Gender	Male = 63	Male = 40	Male = 7	Male = 13	Male = 3
	Female = 24	Female = 19	Female = 1	Female = 3	Female = 1

BMI, body mass index; AS, activity score; CAS, consensus activity score.

### *CYP2D6* phenotypes

The mean MR in the 87 subjects was 4.69 ± 30.8, based on the MR values from the 3 h saliva samples. MR ranged from 0.102 to 288 and had a 2800-fold inter-individual range. The mean MR in the 87 subjects was 1.66 ± 10.5, based on the MR values from the 3 h plasma samples. MR ranged from 0.0266 to 97.9 and had a 3600-fold inter-individual range. The mean urinary MR in the 87 subjects was 0.276 ± 1.41, based on the MR values from the 0–3 h urine samples. MR ranged from 0.00603 to 13.1, and had a 2,100-fold inter-individual range.

**FIGURE 1:**
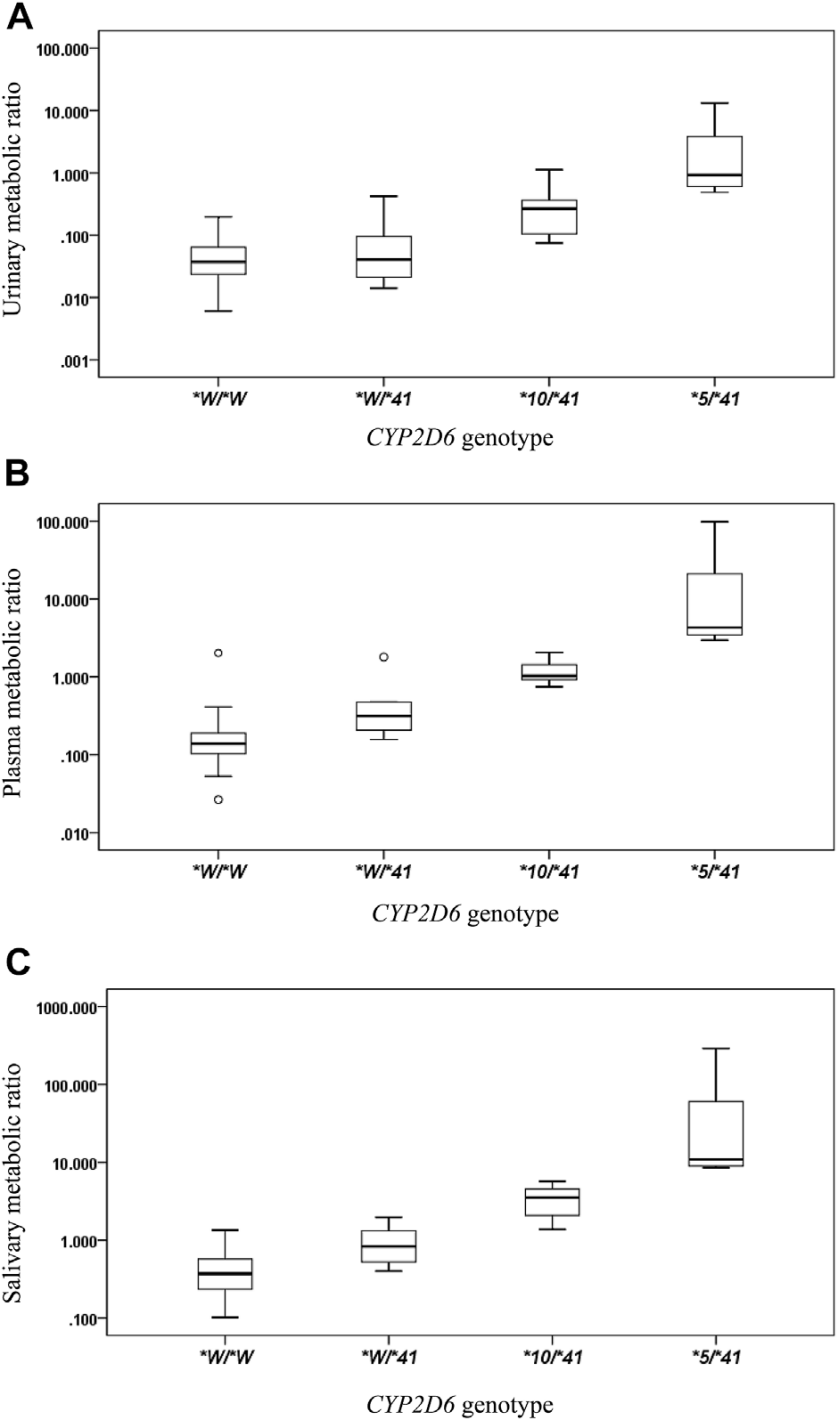
The box plots of metabolic ratio (MR) in the wild type (CYP2D6*1/*1, *1/*2, *2/*2) and the three CYP2D6*41 allele relevant genotypes in 87 healthy Chinese subjects. Allele *W represents CYP2D6*1 or *2. The *y*-axis is logarithmic and the ratio is based on the MR_DM/DX_. **(A)** urinary MR; **(B)** plasma MR; **(C)** salivary MR. Box plot explanation: upper horizontal line of box, 75th percentile; lower horizontal line of box, 25th percentile; horizontal bar within box, median; upper horizontal bar outside box, 95th percentile; lower horizontal bar outside box, 5th percentile. Circles represent outliers.

The CYP2D6*41 allele significantly affected the metabolic activity of *CYP2D6* across all methods used. The mean (±SD) MRs for different Activity Score (AS) or Consensus activity score (CAS) (referring to the consensus CYP2D6 genotype to phenotype translation method in (Caudle et al., 2019)) groups were presented by the three phenotyping methods, respectively (Table 2; Figure 1).

**TABLE 2:** The MRs for different Activity Score (AS) or Consensus Activity Score (CAS) groups based on the three phenotyping methods.

	Group 1 (wild type: CYP2D6*1/*1, *1/*2, and *2/*2) (as = 2, CAS = 2) (n = 59)	Group 2 (CYP2D6*1/*41 and *2/*41) (as = 1.5, CAS = 1.5) (n = 8)	Group 3 (CYP2D6*10/*41) (as = 1, CAS = 0.75) (n = 16)	Group 4 (CYP2D6*5/*41) (as = 0.5, CAS = 0.5) (n = 4)	*p* value (group 1 vs. Group 2)	*p* value (group 2 vs. Group 3)	*p* value (group 3 vs. Group 4)	*p* value (all groups)
Urinary MR	0.0496 ± 0.0364	0.0942 ± 0.136	0.304 ± 0.258	3.87 ± 6.17	0.040	0.044	0.021	<0.001
Plasma MR	0.191 ± 0.256	0.494 ± 0.541	1.21 ± 0.432	27.4 ± 47.0	0.009	0.002	0.025	<0.001
Salivary MR	0.461 ± 0.305	0.971 ± 0.547	3.40 ± 1.56	79.7 ± 138	<0.001	<0.001	0.027	<0.001

AS, activity score; CAS, consensus activity score.

The salivary, plasma, and urinary MRs exhibited a statistically significant increase in subjects with CYP2D6*1(or *2)/*1(or *2), *1 (or *2)/*41, *10/*41, and *5/*41 (all *p* values < 0.05) (Table 2).

### Correlations among urinary, plasma, and salivary MR

**FIGURE 2:**
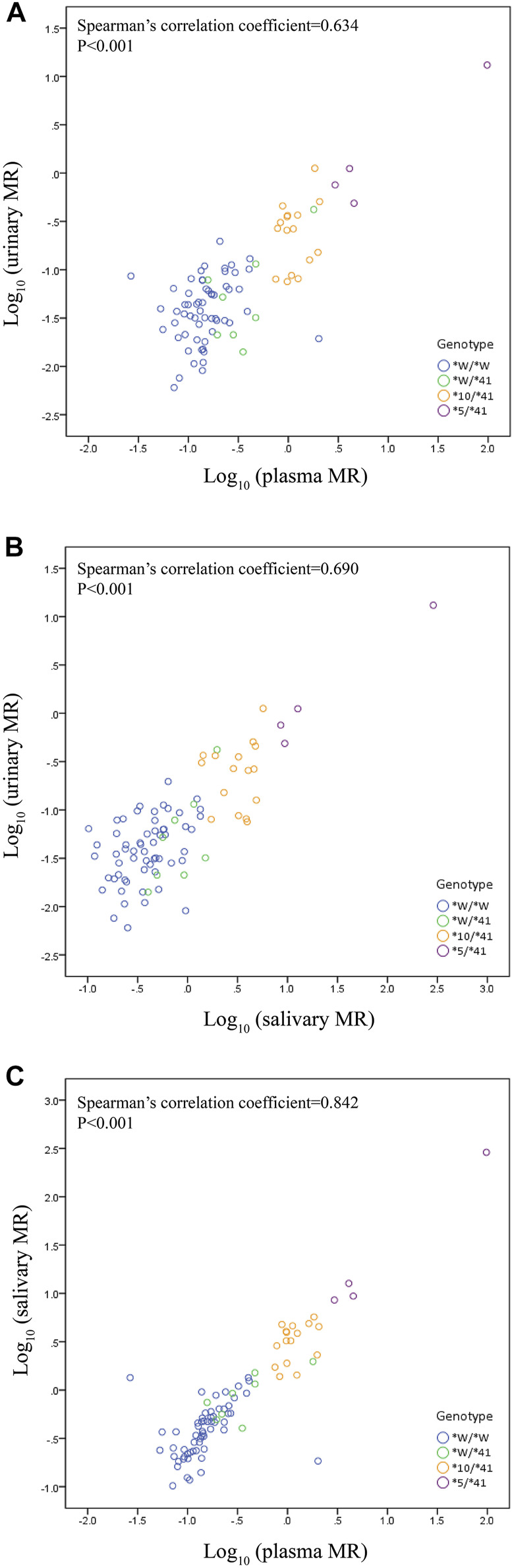
Correlations among urinary, plasma and salivary MRs in the wild type (CYP2D6*1/*1, *1/*2, *2/*2) and the three CYP2D6*41 allele relevant genotypes, respectively, in double logarithmic coordinates. Allele *W represents CYP2D6*1 or *2. **(A)** correlation between urinary MR and plasma MR (Spearman’s correlation coefficient = 0.634 with *p* < 0.001); **(B)** correlation between urinary MR and salivary MR (Spearman’s correlation coefficient = 0.690 with *p* < 0.001); **(C)** correlation between plasma MR and salivary MR (Spearman’s correlation coefficient = 0.842 with *p* < 0.001).

We observed a statistically significant correlation between plasma MR and urinary MR. The Spearman’s correlation coefficient assessing the statistical dependence between the two variables was 0.634 (*p* < 0.001) (Figure 2A). A statistically significant correlation was also observed between salivary MR and urinary MR with a Spearman’s correlation coefficient of 0.690 (*p* < 0.001) (Figure 2B). There was also a statistically significant relationship between salivary MR and plasma MR, while the Spearman’s correlation coefficient was 0.842 (*p* < 0.001) (Figure 2C).

## Discussion

The CYP2D6*41 variant is the second or third frequent reduced function allele in Chinese (3–4%) and the CYP2D6*∗*10 variant is the most frequent allele (42.6%) (Qian et al., 2013). As a well-known reduced function allele besides CYP2D6*10, CYP2D6*41 impacts the enzyme activity of *CYP2D6* to a great extent. Significantly, relatively high frequency of both CYP2D6*10 and *41 alleles in Chinese population as well as the large number of subjects included in this study enable the comparison of CYP2D6*41 metabolic ratio to CYP2D6*10 in the same population. Results of this study add to the evidence of decreasing CYP2D6*41 activity score.

Translating *CYP2D6* genotype to metabolizer phenotype is a topic of great interest in the scientific community and is the focus of several CPIC and DPWG guidelines. According to the previous CPIC guideline, the *CYP2D6* AS is translated into a phenotype using the following classification system: individuals with an AS of 0 are PMs, those with a score of 0.5 are IMs, those with a score of 1.0–2.0 are NMs, and those with a score > 2 are UMs (Kevin Hicks et al., 2016). According to the previous DPWG guideline, the *CYP2D6* AS is then translated into a phenotype using another classification system: individuals with an AS of 0 are PMs, those with a score of 0.5–1.0 are IMs, those with a score of 1.5–2.5 are NMs, and those with a score > 2.5 are UMs (Caudle et al., 2019). In the Consensus Activity Score (CAS) system, the translation method has been modified. Individuals with an AS of 0 are PMs, those with a score of 0.25–1.0 are IMs, those with a score of 1.25–2.25 are NMs, and those with a score > 2.25 are UMs (Caudle et al., 2019). No matter what system is used for the genotype to phenotype translation, assigning a score to an allele is of great importance and a challenge, especially for reduced function alleles. The present study was conducted to investigate the impact of the CYP2D6*41 allele on *CYP2D6* metabolic activity. To exclude confounding factors, we defined CYP2D6*1 and *2 as the full functional allele and CYP2D6*5 as the nonfunctional allele. CYP2D6*10, as a typical reduced function allele, was also involved for comparison. All alleles were used as controls to investigate the impact of CYP2D6*41 in different allelic combinations.

Statistically significant increases were observed in the salivary, plasma, and urinary MR values in subjects with CYP2D6*1(or *2)/*1(or *2), *1 (or *2)/*41, *10/*41, and *5/*41 (all *p* values < 0.05). This result means that as compared with the wild type, one CYP2D6*41 allele combined with one full function allele will significantly reduce the enzyme activity. Likewise, one CYP2D6*41 allele combined with one reduced function allele and one CYP2D6*41 allele combined with one nonfunctional allele will reduce the enzyme activity further and further with statistical significance.

According to the DPWG guideline or consensus activity score system, CYP2D6*10/*41 and *5/*41 are both classified as IMs. However, the MR values of the two groups were significantly different. In addition, in the both systems, group 1 (CYP2D6*1/*1, *1/*2, and *2/*2) and group 2 (CYP2D6*1/*41 and *2/*41) are both classified as NMs, while the MR values of the two groups were significantly different. According to the CPIC guideline, group 1, 2, and 3 are all classified as NMs, while the MR values in these three groups could be more than ten times different with statistical significance. In the current guidelines or translation systems of *CYP2D6*, the definition of NMs or IMs seems to be not reasonably accurate or predictable. The activity score system or the consensus activity score system, instead of the traditional phenotype classification, could predict the *CYP2D6* enzyme activity more accurately.

**FIGURE 3:**
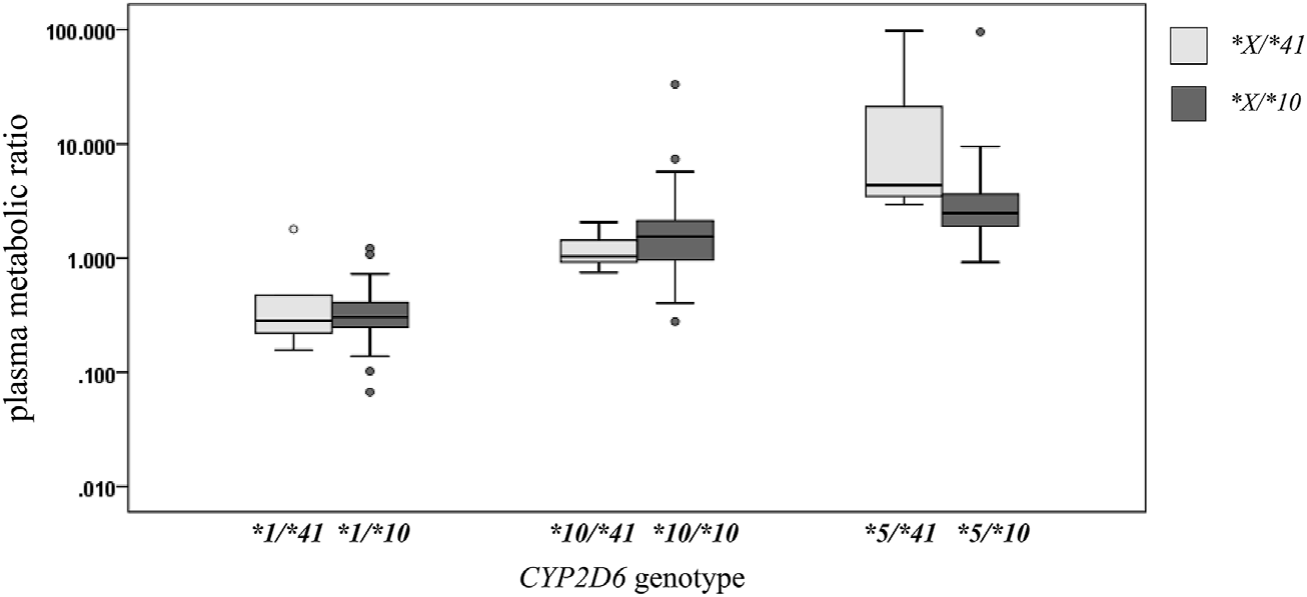
The box plot of comparison of plasma MRs for CYP2D6*1/*41 vs. *1/*10, CYP2D6*10/*41 vs. *10/*10, and CYP2D6*5/*41 vs. *5/*10 genotypes in the subjects. The legend *X/*41 represents genotype *1/*41, *10/*41 or *5/*41. The legend *X/*10 represents genotype *1/*10, *10/*10 or *5/*10.

Assigning a score to an allele is important and a challenge for CYP2D6*41. In a previous study with the similar study design (Chen et al., 2017), the urinary, plasma, or salivary MRs in subjects with CYP2D6*1/*10 were reported to be 0.0915, 0.351, and 1.05, respectively. The urinary, plasma, or salivary MRs in subjects with CYP2D6*10/*10 were reported to be 0.420, 2.03, and 5.09, respectively. The urinary, plasma, or salivary MRs in subjects with CYP2D6*5/*10 were reported to be 1.96, 5.63, and 19.8, respectively (data were presented in the previous study). The comparison between subjects with CYP2D6*1/*41 and those with CYP2D6*1/*10 showed the MRs in the two groups were quite close. The MRs between subjects with CYP2D6*10/*41 and CYP2D6*10/*10 were different but within two-fold. The MRs of subjects with CYP2D6*5/*41 were generally larger than the MRs of those with CYP2D6*5/*10 (with no significance) (Table 3; Figure 3). According to the results based on the limited sample size, CYP2D6*41 had similar impact on the *CYP2D6* enzyme activity as compared with CYP2D6*10, or CYP2D6*41 reduced the enzyme activity more than CYP2D6*10. In no matter which case, assigning *41 a score of 0.5 and assigning *10 a score of 0.25 according to the consensus AS system could be reconsidered. However, when comparing enzymatic activity of different *CYP2D6* alleles and evaluating their activity score, an indispensable consideration is the substrate specificity. Changing study substrate might come to opposite conclusions (Steimer et al., 2004; Hicks et al., 2014). Further clinical studies would be needed to provide more information.

**TABLE 3:** The plasma MR comparisons between CYP2D6*1/*41 vs. *1/*10, *10/*41 vs. *10/*10, and *5/*41 vs. *5/*10.

	CYP2D6*1/*X		CYP2D6*10/*X		CYP2D6*5/*X	
	CYP2D6*1/*41 (n = 5)	CYP2D6*1/*10 (n = 93)	CYP2D6*10/*41 (n = 16)	CYP2D6*10/*10 (n = 85)	CYP2D6*5/*41 (n = 4)	CYP2D6*5/*10 (n = 35)
Plasma MR	0.586 ± 0.688	0.351 ± 0.180	1.21 ± 0.432	2.02 ± 3.57	27.4 ± 47.0	5.63 ± 15.7
*p* value	0.488^a^	0.365	0.424^a^			

a Analyzed with Welch’s ANOVA, due to heterogeneity of variances of data.

The correlation between the MRs from 0–3 h urine, 3 h single-point plasma, and 3 h single-point saliva were reported to be good in subjects with wild type and *10 relevant genotypes of *CYP2D6* in the previous study (Chen et al., 2017). In the present study, the correlation between the MRs from the three kinds of samples were demonstrated to be good in subjects with wild type and *41 relevant genotypes of *CYP2D6*. These two studies found that salivary, plasma, and urinary MRs displayed a high correlation, and that different phenotyping techniques can be used in clinical settings. This result would be useful for clinical practice. For a long time, urine sample collection for 8 h after dosing is the standard process for phenotyping patients, while it was quite a demanding process in clinical practice, especially for outpatients who had to deliver the urinary samples at a later time. Single-point plasma and salivary phenotyping methods would save much time and be more convenient for clinical practice. Other previous studies also indicated that MR from single-point plasma from 1 to 30 h after a single dose of DM could predict the MR from AUC well and could be used as the *CYP2D6* phenotyping method for NMs, IMs, and PMs (Chen et al., 2016a; Chen et al., 2016b). The result was consistent with data previously reported from small samples (Chladek et al., 2000; Frank et al., 2007).

While their absolute values might be different, the DM/total-DX ratio and the DM/free-DX ratio can both be used to phenotype *CYP2D6*. As same as in the previous study (Chen et al., 2017), only unconjugated DX was observed in saliva, while the DM/free-DX ratio was in each of this study’s three sample types. As such, the accepted anti-mode of DM/total-DX of 0.3 between NMs and PMs did not apply to this study. We observed significant overlaps of MR values between similar genotypes and it was always the challenge when predicting phenotypes from *CYP2D6* genotypes. It was difficult to explain some outliers observed in the present study, and therefore there was inevitable uncertainty as to whether an individual with a certain allele would express the predicted phenotype (Leeder and Gaedigk, 2014). Particularly, RNA level or protein level is a closer indicator to phenotype than genotype. Recent study suggests liquid biopsy provides a practical way to quantify cytochrome P450 expression level which correlates well to metabolic activity. This promising technique may contribute to precision dosing therapy (Achour et al., 2022).

## Conclusion

In subjects with CYP2D6*1(or *2)/*1(or *2), *1 (or *2)/*41, *10/*41, and *5/*41, the salivary, plasma, or urinary MR values increased successively (all *p* < 0.001). The activity score system or the consensus activity score system, instead of the traditional phenotype classification, could predict the *CYP2D6* enzyme activity more accurately. CYP2D6*41 had similar or more impact on the *CYP2D6* enzyme activity as compared with CYP2D6*10. Assigning *41 a score of 0.5 and assigning *10 a score of 0.25 according to the consensus AS system should be reconsidered. Alternative techniques for phenotyping are saliva and single-point plasma, which provide significant clinical convenience.

## Data Availability

The datasets presented in this article are not readily available due to privacy and ethical restrictions. Requests to access the datasets should be directed to the corresponding authors.
